# Antimicrobial Susceptibility Profile of Klebsiella pneumoniae Isolates From a Tertiary Care Hospital: A Retrospective Study

**DOI:** 10.7759/cureus.94006

**Published:** 2025-10-07

**Authors:** Kumudini Panigrahi, Basanti Kumari Pathi, Jyoti Prakash Sahoo

**Affiliations:** 1 Microbiology, Kalinga Institute of Medical Sciences, Bhubaneswar, IND; 2 Pharmacology, Kalinga Institute of Medical Sciences, Bhubaneswar, IND

**Keywords:** antibiotic sensitivity and resistance, antimicrobial resistance, antimicrobial susceptibility testing, bacterial biofilm, community-acquired infection, extended spectrum β-lactamase, hospital-acquired infection, intensive care unit, klebsiella pneumoniae (kp), multidrug-resistant organism (mdro)

## Abstract

Background and objectives: Globally, drug resistance has been growing in number and severity. *Klebsiella pneumoniae* is one of the multidrug-resistant organisms (MDROs) found in healthcare settings, especially in intensive care units (ICUs). We mapped this study to determine the antimicrobial susceptibility testing (AST) patterns of *K. pneumoniae* isolates found in our hospital. We also determined the AST findings of the ICU and non-ICU patients and those with hospital-acquired infection (HAI) and community-acquired infection (CAI).

Methods: This retrospective study was conducted from October 2022 to September 2024 at Kalinga Institute of Medical Sciences (KIMS), Bhubaneswar, India. We included adult inpatients with positive culture reports for *K. pneumoniae*. All specimens (blood, urine, endotracheal tube (ETT), body fluid, sputum, pus, wound, and skin swabs) from the eligible participants were analysed for their AST findings. The subgroup analyses of the AST findings were performed based on the location (ICU or non-ICU) and infection type (HAI or CAI). We presented the findings through a mosaic plot and a chord diagram. We employed the VITEK 2 system for AST. R software (version 4.4.3) was leveraged for data analysis.

Results: We assessed 7,942 different samples in our study. The study population comprised 2,994 (37.70%) females and 4,948 (62.30%) males. The median age was 53.50 (39.75-63.25) years. The majority of samples were obtained from blood (2591, 32.62%), urine (1975, 24.87%), ETT (1887, 23.76%), and body fluid (462, 5.82%). A total of 3,523 (44.36%) participants were hospitalized in ICUs. This study's CAI and HAI cases were 1,541 (19.40%) and 6,401 (80.60%). Among the 7,942 *K. pneumoniae* isolates found among all participants, the maximum susceptibility was observed towards tigecycline (5,940, 74.79%), followed by gentamicin (3,133, 39.45%), meropenem (2,727, 34.34%), and piperacillin-tazobactam (2,576, 32.44%). The resistance was the highest against ciprofloxacin (6,227, 78.40%), followed by cefuroxime (5,809, 73.14%), and ceftriaxone (5,397, 67.96%). The resistance was the lowest against tigecycline (999, 12.57%). The subgroup analyses were similar.

Conclusion: Blood, urine, and ETT specimens contributed most of the samples. In our study, HAI was four times more common than CAI. *K. pneumoniae* isolates showed good sensitivity for tigecycline, gentamicin, meropenem, and piperacillin-tazobactam. The resistance was higher against ciprofloxacin, cefuroxime, and ceftriaxone than against other antibiotics.

## Introduction

Antimicrobial resistance (AMR) is a worldwide threat that challenges breakthroughs across multiple disciplines [1]. Among the Enterobacterales, *Klebsiella pneumoniae* is one of the most prevalent multidrug-resistant organisms (MDROs) [1,2]. *Klebsiella* species are rod-shaped, nonmotile, Gram-negative bacteria. These are commensals of the mouth, nasopharynx, and gastrointestinal system in humans [3,4]. Pneumonia, urinary tract infections (UTIs), meningitis, skin and soft tissue infections, liver abscesses, and endophthalmitis are the common diseases caused by *Klebsiella* species [3,5]. *Enterococcus faecium*, *Staphylococcus aureus*, *Klebsiella pneumoniae*, *Acinetobacter baumannii*, *Pseudomonas aeruginosa*, and *Enterobacter *spp (ESKAPE) pathogens cause the majority of the hospital-acquired infections (HAIs). *K. pneumoniae* is the "K" in that group [6,7]. Globally, most drug-resistant infections are brought on by extended-spectrum beta-lactamase (ESBL) and carbapenemase-producing *K. pneumoniae* [7-9].

Cell wall protein receptors and lipopolysaccharides (LPS) contribute to the pathogenicity of *K. pneumoniae*. These elements mediate the binding process to host cells and enable protection against the human immune system [10]. The antibiotic resistance of bacteria is grouped into the following three categories: multidrug-resistant (MDR), extensively drug-resistant (XDR), and pandrug-resistant (PDR). MDR *K. pneumoniae* develops resistance towards at least one antimicrobial from three different drug classes. XDR *K. pneumoniae* strains are resistant to at least one antibiotic from most antibiotic classes. PDR *K. pneumoniae* isolates are resistant to each drug in all antibiotic groups [11-13]. The nosocomial infections by *K. pneumoniae* are increasing in the ICUs [10,14]. Moreover, the MDR *K. pneumoniae* strains are most frequently seen in the ICUs [10,15].

Community-acquired infection (CAI) can be described as an infection either incubating or present during hospitalization or within 48 hours of admission. The infection that develops after 48 hours of hospitalization is regarded as HAI [16,17]. Nowadays, *K. pneumoniae* is one of the leading causes of HAI and CAI [18-22]. Recently, Sharma et al. [23] and Kaur et al. [24] discovered new trends in antimicrobial susceptibility testing (AST) findings of *K. pneumoniae* isolates in India. We planned this study to evaluate the prevalence of infections caused by *K. pneumoniae* in our hospital and the AST findings of those bacterial isolates. We also determined the AST findings of the *K. pneumoniae* isolates of ICU and non-ICU patients and those with HAI and CAI.

## Materials and methods

Study design

This retrospective study was conducted between October 2022 and September 2024 at Kalinga Institute of Medical Sciences (KIMS), Bhubaneswar, India. The Institutional Ethics Committee of Kalinga Institute of Medical Sciences (KIMS), Bhubaneswar, India, granted us ethical clearance to begin the study (KIIT/KIMS/IEC/1917/2024, dated 23.10.2024). The study complied with the Strengthening the Reporting of Observational Studies in Epidemiology (STROBE) guideline, the Declaration of Helsinki, Good Laboratory Practices, and institutional norms.

Patient recruitment

We scrutinized the laboratory data of the adult patients having positive culture reports who got admitted to our hospital during the above-mentioned period. Only those patients who had positive *K. pneumoniae* culture reports were analysed. The AST findings of those isolates were assessed. Inadequate samples and outpatients were excluded.

Data collection

Blood, endotracheal tube (ETT), body fluid, and urine samples were collected and sent to the microbiological laboratory. Following Gram staining, respiratory and body fluid samples were inoculated into blood agar and MacConkey agar. Then they were incubated overnight with 5% CO_2_ in an incubator. Blood samples were incubated within the BacT/Alert 3D machine (bioMérieux, Marcy-l'Étoile, France). They were cultivated on MacConkey agar and sheep blood agar following a positive flag. Urine samples were inoculated on cystine-lactose-electrolyte deficient (CLED) agar. MacConkey agar and blood agar were used to inoculate other specimens, e.g., pus, wound swab, nasal swab, and skin swab. All of the samples were incubated for 24-48 hours at 37°C. Strains of *K. pneumoniae* were distinguished by their biochemical and morphological traits. Using the Clinical and Laboratory Standard Institute (CLSI) 2022 cut-off values [25], the VITEK 2 system (bioMérieux, Marcy-l'Étoile, France) was utilized to identify isolates and assess antimicrobial susceptibility. AST functions through micro-broth dilution. The term "minimum inhibitory concentration" (MIC) refers to the highest dilution of an antibiotic that inhibits the growth of the organism. Validated software was used to interpret the growth kinetics and MIC data of *K. pneumoniae* isolates. All data were collected from the microbiology laboratory and analysed.

Statistical analysis

This retrospective study was accomplished using convenience sampling. All patients admitted between October 2022 and September 2024 were scrutinized. Those having positive culture reports for *K. pneumoniae* were considered for analysis. The Kolmogorov-Smirnov test was used to ascertain the normality of the data distribution. We calculated the continuous data's median and interquartile range (IQR). For the categorical data, we computed the frequency and proportion. We created a mosaic plot to show the participants' distribution. Clinical samples (blood, ETT, urine, body fluids, or others), location (ICU or non-ICU), infection type (HAI or CAI), and medication resistance status (MDR or non-MDR) were the parameters used for preparing the mosaic plot. The AST patterns of *K. pneumoniae* isolates from the entire research population and different subgroups were shown using chord diagrams. The R Program (Vienna, Austria) version 4.4.3 was used for data analysis and plot generation [26]. p-values less than 0.05 were interpreted as statistically significant.

## Results

We screened only the 50,057 culture-positive reports of the patients admitted during the stipulated duration. Out of 13,547 positive blood samples, 2,591 (19.13%) were positive for *K. pneumoniae*. A total of 1,975 (11.17%) of 17,683 urine samples and 1,887 (30.27%) of 6,234 ETT samples were positive for *K. pneumoniae*. Of 4,971 positive body fluid samples, 462 (9.29%) were positive for *K. pneumoniae*. A total of 1,027 (13.47%) of the other 7,622 positive samples were sputum, nasal swab, pus, bronchoalveolar lavage (BAL), wound, and skin samples. The study profile is shown in Table 1. The median age of the participants was 53.50 (39.75-63.25) years. Our study had 4,948 (62.30%) male patients. Of the 7,942 positive samples collected, the most common sample was blood (2,591, 32.62%), followed by urine (1,975, 24.87%), ETT (1,887, 23.76%), and body fluid (462, 5.82%). Other samples, like sputum, nasal swabs, pus, bronchoalveolar lavage (BAL), wound, and skin samples, comprised 1,027 (12.93%) of all positive samples. A total of 3,523 (44.36%) participants were admitted to the ICU. The incidences of CAI and HAI in our hospital were 1,541 (19.40%) and 6,401 (80.60%). MDR cases were 4,418 (55.63%).

**Table 1:** Demographics of the study population The continuous variables were expressed as median and IQR. The categorical variables were expressed as frequency and percentage. IQR: interquartile range, ETT: endotracheal tube, ICU: intensive care unit, CAI: community-acquired infection, HAI: hospital-acquired infection, MDR: multidrug resistance.

Parameter	Value
Total participants	7,942
Age (years)	53.50 (39.75-63.25)
Male	4,948 (62.30%)
Samples collected (positive for *Klebsiella pneumoniae*)	
ETT	1,887 (23.76%)
Blood	2,591 (32.62%)
Body fluid	462 (5.82%)
Urine	1,975 (24.87%)
Others	1,027 (12.93%)
Location	
ICU	3,523 (44.36%)
Non-ICU	4,419 (55.64%)
Infection type	
CAI	1,541 (19.40%)
HAI	6,401 (80.60%)
Drug resistance status	
MDR	4,418 (55.63%)
Non-MDR	3,524 (44.37%)

Distribution of the study population

**Figure 1:**
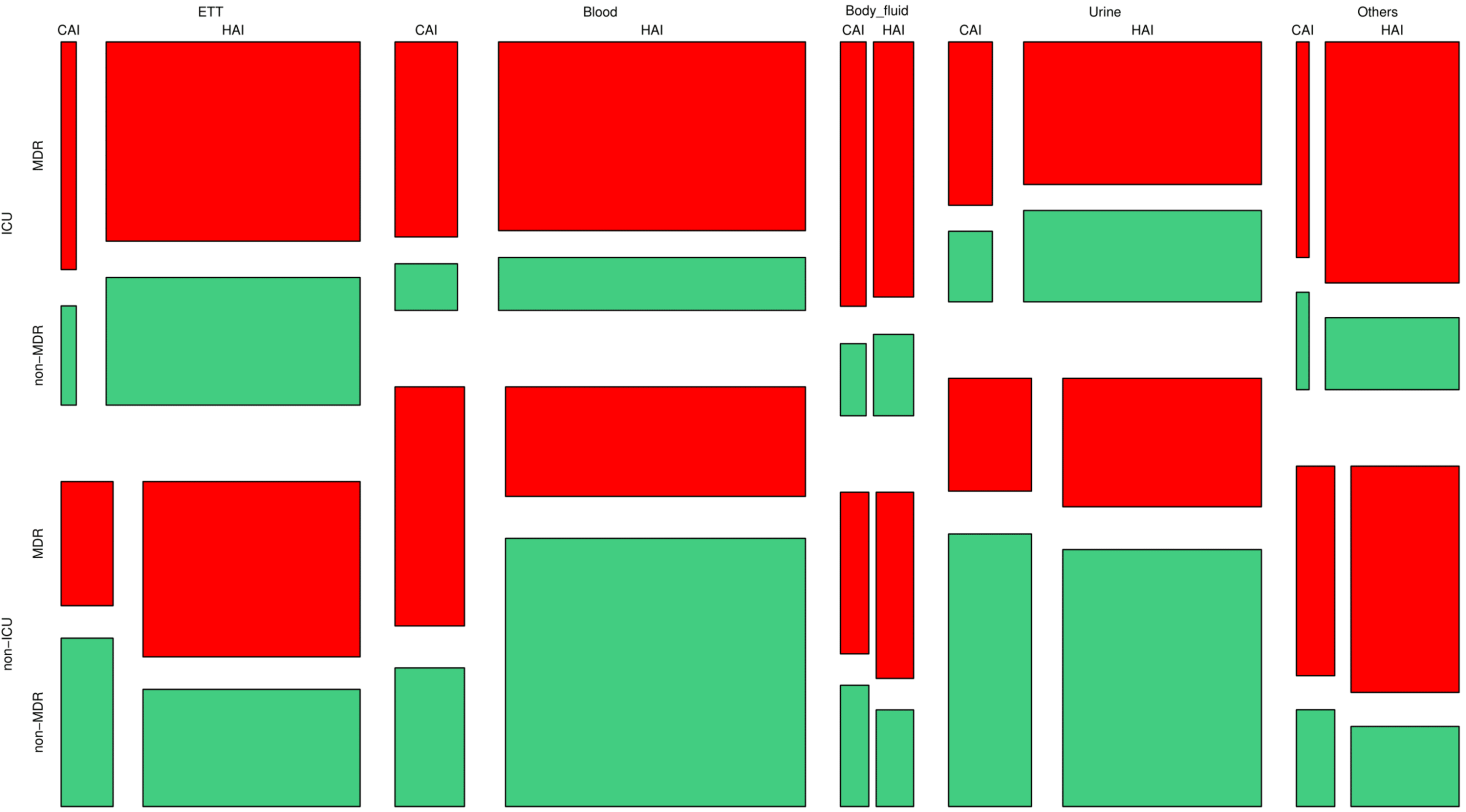
Distribution of the study population The mosaic plot showcases the study participants’ distribution per the sample used (ETT, blood, body fluid, urine, or others), location (ICU or non-ICU), infection type (CAI or HAI), and drug resistance status (MDR or non-MDR). ETT: endotracheal tube, ICU: intensive care unit, CAI: community-acquired infection, HAI: hospital-acquired infection, MDR: multidrug resistance.

Figure 1 shows the participants’ distribution via a mosaic plot. We segregated the study population with the following parameters: sample used (ETT, blood, body fluid, urine, or others), location (ICU or non-ICU), infection type (CAI or HAI), and drug resistance status (MDR or non-MDR). The order of the division was sample collected, location, infection type, and resistance status. The most frequent samples (positive for *K. pneumoniae*) were obtained from blood (2,591, 32.62%), followed by urine (1,975, 24.87%), ETT (1,887, 23.76%), and body fluid (462, 5.82%). The largest block represents the non-ICU patients with HAI and blood culture positive for non-MDR *K. pneumoniae* (911, 11.47%), followed by the ICU patients with HAI and blood culture positive for MDR *K. pneumoniae* (656, 8.26%), the non-ICU patients with HAI and urine culture positive for non-MDR *K. pneumoniae* (578, 7.28%), and the ICU patients with HAI and ETT culture positive for MDR *K. pneumoniae* (573, 7.21%).

AST patterns of the study population and various subgroups

**Figure 2:**
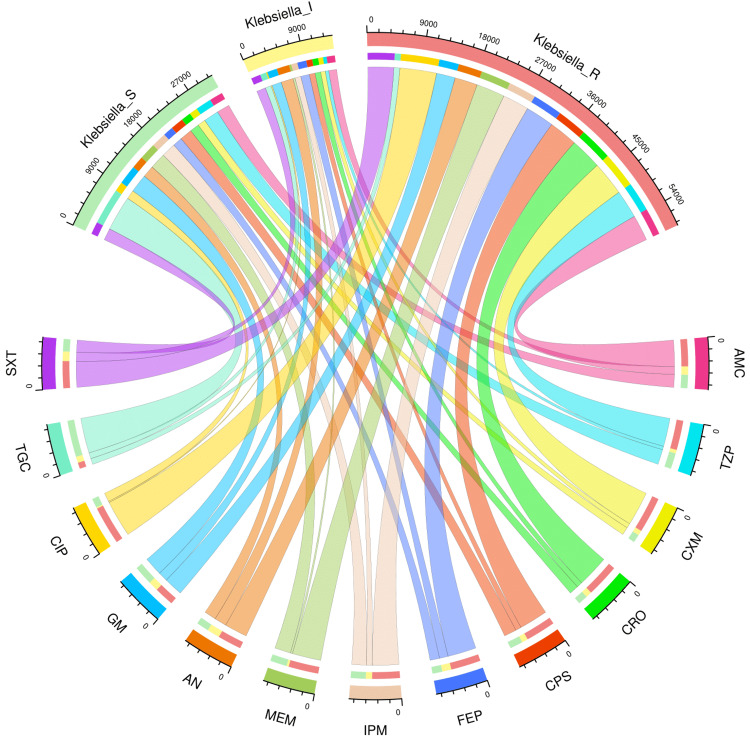
AST findings of Klebsiella pneumoniae isolates found among the study participants (n = 7,942) The lower and upper sections represent 13 drugs (shown in different colours) and three types of antibiotic susceptibility (S: sensitive, I: intermediate, and R: resistant) of *Klebsiella pneumoniae* isolates found in the entire study population (n = 7,942). The widths of the bands correspond with the number of Klebsiella pneumoniae isolates and their AST patterns for the 13 drugs. AST: antimicrobial sensitivity testing, AMC: amoxicillin-clavulanic acid, TZP: piperacillin-tazobactam, CXM: cefuroxime, CRO: ceftriaxone, CPS: cefoperazone-sulbactam, FEP: cefepime, IPM: imipenem, MEM: meropenem, AN: amikacin, GM: gentamicin, CIP: ciprofloxacin, TGC: tigecycline, SXT: cotrimoxazole.

**Figure 3:**
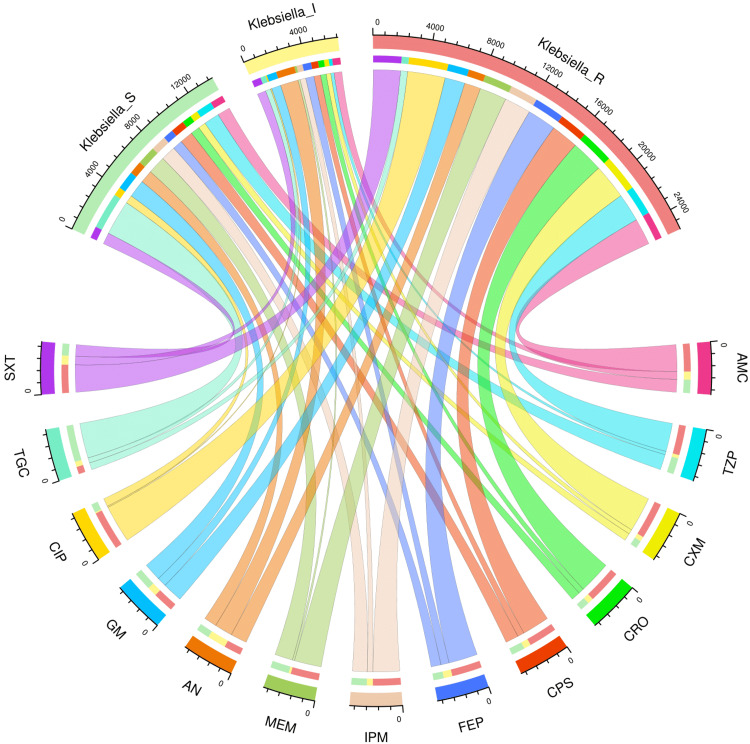
AST findings of Klebsiella pneumoniae isolates found among the ICU patients (n = 3,523) The lower and upper sections represent 13 drugs (shown in different colours) and three types of antibiotic susceptibility (S: sensitive, I: intermediate, and R: resistant) of *Klebsiella pneumoniae* isolates found in the ICU patients (n = 3,523). The widths of the bands correspond with the number of *Klebsiella pneumoniae* isolates and their AST patterns for the 13 drugs. ICU: intensive care unit, AST: antimicrobial sensitivity testing, AMC: amoxicillin-clavulanic acid, TZP: piperacillin-tazobactam, CXM: cefuroxime, CRO: ceftriaxone, CPS: cefoperazone-sulbactam, FEP: cefepime, IPM: imipenem, MEM: meropenem, AN: amikacin, GM: gentamicin, CIP: ciprofloxacin, TGC: tigecycline, SXT: cotrimoxazole.

**Figure 4:**
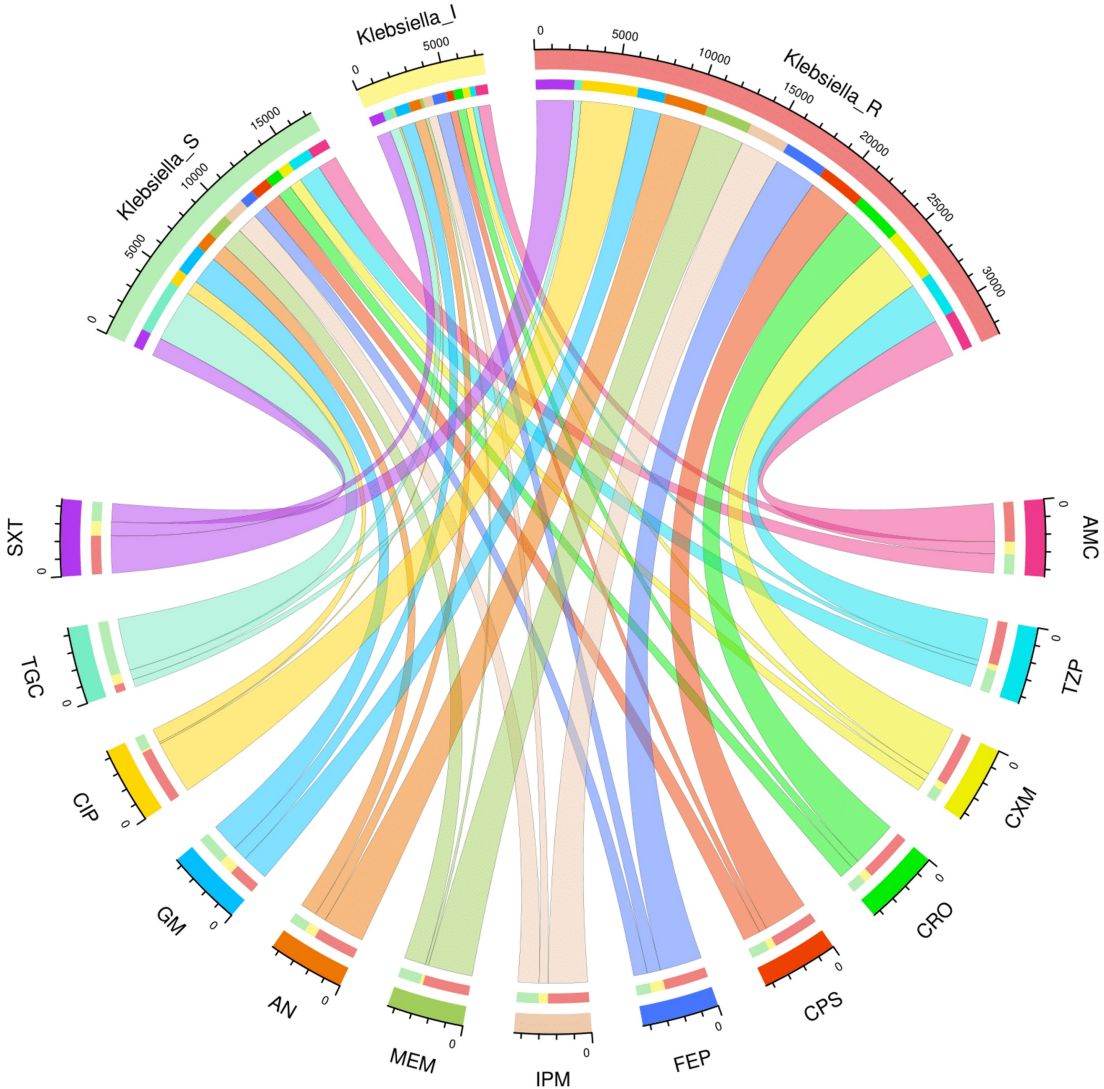
AST findings of Klebsiella pneumoniae isolates found among the non-ICU patients (n = 4,419) The lower and upper sections represent 13 drugs (shown in different colours) and three types of antibiotic susceptibility (S: sensitive, I: intermediate, and R: resistant) of *Klebsiella pneumoniae* isolates found in the non-ICU patients (n = 4,419). The widths of the bands correspond with the number of *Klebsiella pneumoniae* isolates and their AST patterns for the 13 drugs. ICU: intensive care unit, AST: antimicrobial sensitivity testing, AMC: amoxicillin-clavulanic acid, TZP: piperacillin-tazobactam, CXM: cefuroxime, CRO: ceftriaxone, CPS: cefoperazone-sulbactam, FEP: cefepime, IPM: imipenem, MEM: meropenem, AN: amikacin, GM: gentamicin, CIP: ciprofloxacin, TGC: tigecycline, SXT: cotrimoxazole.

**Figure 5:**
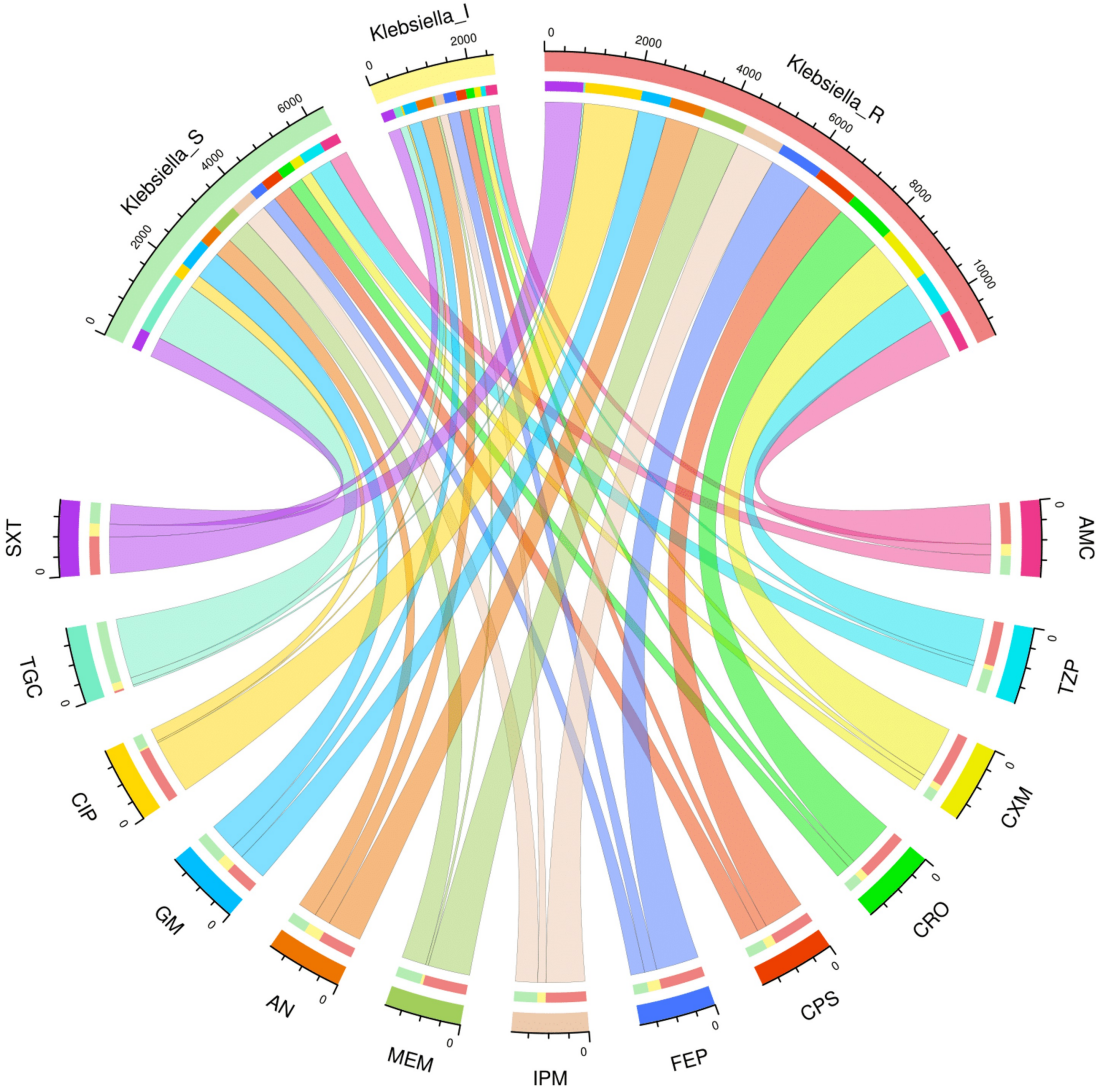
AST findings of Klebsiella pneumoniae isolates found among the patients with CAI (n = 1,541) The lower and upper sections represent 13 drugs (shown in different colours) and three types of antibiotic susceptibility (S: sensitive, I: intermediate, and R: resistant) of *Klebsiella pneumoniae* isolates found in the patients with CAI (n = 1,541). The widths of the bands correspond with the number of *Klebsiella pneumoniae* isolates and their AST patterns for the 13 drugs. CAI: community-acquired infection, AST: antimicrobial sensitivity testing, AMC: amoxicillin-clavulanic acid, TZP: piperacillin-tazobactam, CXM: cefuroxime, CRO: ceftriaxone, CPS: cefoperazone-sulbactam, FEP: cefepime, IPM: imipenem, MEM: meropenem, AN: amikacin, GM: gentamicin, CIP: ciprofloxacin, TGC: tigecycline, SXT: cotrimoxazole.

**Figure 6:**
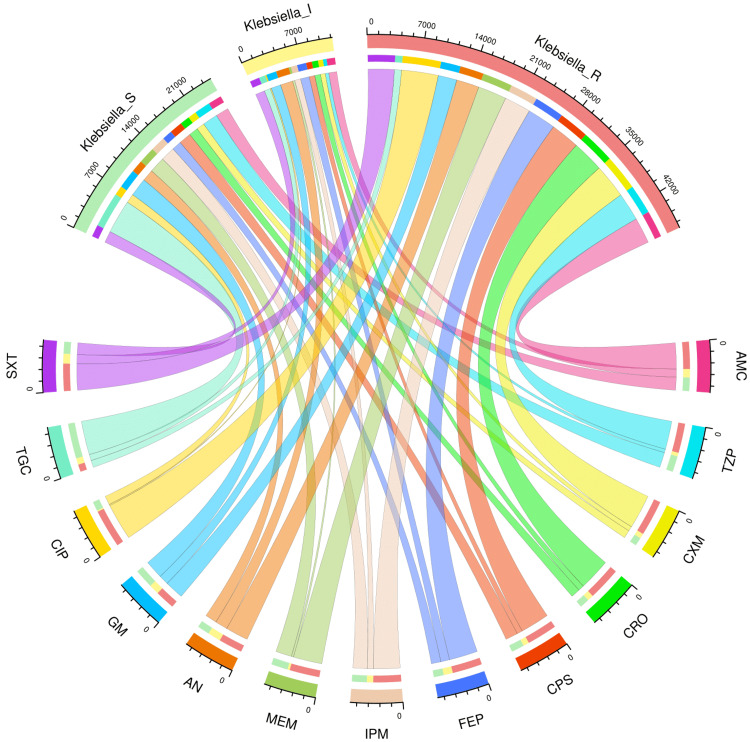
AST findings of Klebsiella pneumoniae isolates found among the patients with HAI (n = 6,401) The lower and upper sections represent 13 drugs (shown in different colours) and three types of antibiotic susceptibility (S: sensitive, I: intermediate, and R: resistant) of *Klebsiella pneumoniae* isolates found in the patients with HAI (n = 6,401). The widths of the bands correspond with the number of *Klebsiella pneumoniae* isolates and their AST patterns for the 13 drugs. HAI: hospital-acquired infection, AST: antimicrobial sensitivity testing, AMC: amoxicillin-clavulanic acid, TZP: piperacillin-tazobactam, CXM: cefuroxime, CRO: ceftriaxone, CPS: cefoperazone-sulbactam, FEP: cefepime, IPM: imipenem, MEM: meropenem, AN: amikacin, GM: gentamicin, CIP: ciprofloxacin, TGC: tigecycline, SXT: cotrimoxazole.

Figures 2-6 demonstrate the AST patterns of *K. pneumoniae* isolates via chord diagrams. Among the 7,942 *K. pneumoniae* isolates detected in our study population (Figure 2), the highest sensitivity was seen towards tigecycline (5,940, 74.79%), followed by gentamicin (3,133, 39.45%), meropenem (2,727, 34.34%), and piperacillin-tazobactam (2,576, 32.44%). The resistance was the maximum against ciprofloxacin (6,227, 78.40%), followed by cefuroxime (5,809, 73.14%), and ceftriaxone (5,397, 67.96%). The drug resistance was the least against tigecycline (999, 12.57%). Among the 3,523 *K. pneumoniae* isolates detected in our ICU participants (Figure 3), the highest sensitivity was seen towards tigecycline (2,618, 74.31%), followed by meropenem (1,345, 38.18%), gentamicin (1,325, 37.61%), and piperacillin-tazobactam (1,161, 32.95%). The resistance was the maximum against ciprofloxacin (2,821, 80.07%), followed by cefuroxime (2,614, 74.20%), and ceftriaxone (2,422, 68.75%). The drug resistance was the least against tigecycline (521, 14.79%). Among the 4,419 *K. pneumoniae* isolates detected in our non-ICU participants (Figure 4), the highest sensitivity was seen towards tigecycline (3,322, 75.18%), followed by gentamicin (1,808, 40.91%), piperacillin-tazobactam (1,415, 32.02%), meropenem (1,382, 31.27%), and imipenem (1,337, 30.26%). The resistance was the maximum against ciprofloxacin (3,406, 77.08%), followed by cefuroxime (3,195, 72.30%), and ceftriaxone (2,975, 67.32%). The drug resistance was the least against tigecycline (478, 10.82%). Among the 1,541 *K. pneumoniae isolates* detected in the participants with CAI (Figure 5), the highest sensitivity was seen towards tigecycline (1,333, 86.50%), followed by gentamicin (621, 40.23%), meropenem (545, 35.37%), piperacillin-tazobactam (498, 32.32%), and cotrimoxazole (447, 29.01%). The resistance was the maximum against ciprofloxacin (1,211, 78.59%), followed by cefuroxime (1,149, 74.56%), and ceftriaxone (1,051, 68.20%). The drug resistance was the least against tigecycline (42, 2.73%). Among the 6,401 *K. pneumoniae* isolates detected in the participants with HAI (Figure 6), the highest sensitivity was seen towards tigecycline (4,607, 71.97%), followed by gentamicin (2,512, 39.24%), meropenem (2,182, 34.09%), and piperacillin-tazobactam (2,078, 32.46%). The resistance was the maximum against ciprofloxacin (5,016, 78.36%), followed by cefuroxime (4,660, 72.80%), and ceftriaxone (4,346, 67.90%). The drug resistance was the least against tigecycline (957, 14.95%). Table 2 narrates the frequencies and proportions of all *K. pneumoniae* specimens and their AST findings.

**Table 2:** AST findings of Klebsiella pneumoniae isolates AST findings of *Klebsiella pneumoniae* isolates are presented as numbers and percentages. AST: antimicrobial susceptibility testing, ICU: intensive care unit, CAI: community-acquired infection, HAI: hospital-acquired infection, AMC: amoxicillin-clavulanic acid, TZP: piperacillin-tazobactam, CXM: cefuroxime, CRO: ceftriaxone, CPS: cefoperazone-sulbactam, FEP: cefepime, IPM: imipenem, MEM: meropenem, AN: amikacin, GM: gentamicin, CIP: ciprofloxacin, TGC: tigecycline, SXT: cotrimoxazole.

Susceptibility type	AMC	TZP	CXM	CRO	CPS	FEP	IPM	MEM	AN	GM	CIP	TGC	SXT
Total (n = 7,942)													
Sensitive	2,178 (27.42%)	2,576 (32.44%)	1,342 (16.90%)	1,577 (19.86%)	2,099 (26.43%)	1,623 (20.44%)	2,444 (30.77%)	2,727 (34.34%)	2,017 (25.40%)	3,133 (39.45%)	1,516 (19.08%)	5,940 (74.79%)	2,022 (25.46%)
Intermediate	1,349 (16.99%)	581 (7.32%)	791 (9.96%)	968 (12.19%)	978 (12.31%)	1,471 (18.52%)	1,006 (12.67%)	351 (4.42%)	2,061 (25.95%)	1,616 (20.35%)	199 (2.50%)	1,003 (12.63%)	1,544 (19.44%)
Resistant	4,415 (55.59%)	4,785 (60.25%)	5,809 (73.14%)	5,397 (67.96%)	4,865 (61.26%)	4,848 (61.04%)	4,492 (56.56%)	4,864 (61.25%)	3,864 (48.65%)	3,193 (40.20%)	6,227 (78.40%)	999 (12.57%)	4,376 (55.10%)
ICU (n = 3,523)													
Sensitive	951 (26.99%)	1,161 (32.95%)	564 (16.01%)	677 (19.22%)	975 (27.68%)	745 (21.15%)	1,107 (31.42%)	1,345 (38.18%)	955 (27.11%)	1,325 (37.61%)	611 (17.34%)	2,618 (74.31%)	853 (24.21%)
Intermediate	583 (16.55%)	243 (6.90%)	345 (9.79%)	424 (12.04%)	504 (14.31%)	618 (17.54%)	428 (12.15%)	159 (4.51%)	1,345 (38.18%)	700 (19.87%)	91 (2.58%)	384 (10.90%)	648 (18.39%)
Resistant	1,989 (56.46%)	2,119 (60.15%)	2,614 (74.20%)	2,422 (68.75%)	2,044 (58.02%)	2,160 (61.31%)	1,988 (56.43%)	2,019 (57.30%)	1,223 (34.71%)	1,498 (42.52%)	2,821 (80.07%)	521 (14.79%)	2,022 (57.39%)
Non-ICU (n = 4,419)													
Sensitive	1,227 (27.76%)	1,415 (32.02%)	778 (17.60%)	900 (20.37%)	1,124 (25.44%)	878 (19.87%)	1,337 (30.26%)	1,382 (31.27%)	1,062 (24.03%)	1,808 (40.91%)	905 (20.48%)	3,322 (75.18%)	1,169 (26.45%)
Intermediate	766 (17.33%)	338 (7.65%)	446 (10.09%)	544 (12.31%)	474 (10.73%)	853 (19.30%)	578 (13.08%)	192 (4.34%)	716 (16.20%)	916 (20.73%)	108 (2.44%)	619 (14.00%)	896 (20.28%)
Resistant	2,426 (54.90%)	2,666 (60.33%)	3,195 (72.30%)	2,975 (67.32%)	2,821 (63.84%)	2,688 (60.83%)	2,504 (56.66%)	2,845 (64.39%)	2,641 (59.77%)	1,695 (38.36%)	3,406 (77.08%)	478 (10.82%)	2,354 (53.27%)
CAI (n = 1,541)													
Sensitive	409 (26.54%)	498 (32.32%)	251 (16.29%)	317 (20.57%)	419 (27.90%)	313 (20.31%)	495 (32.12%)	545 (35.37%)	421 (27.32%)	621 (40.23%)	291 (18.89%)	1,333 (86.50%)	447 (29.01%)
Intermediate	248 (16.09%)	102 (6.62%)	141 (9.15%)	173 (11.23%)	216 (14.02%)	273 (17.72%)	181 (11.75%)	59 (3.83%)	362 (23.49%)	290 (18.82%)	39 (2.53%)	166 (10.77%)	276 (17.91%)
Resistant	844 (57.35%)	941 (61.06%)	1,149 (74.56%)	1,051 (68.20%)	906 (58.79%)	955 (61.97%)	865 (56.13%)	937 (60.80%)	758 (49.19%)	630 (40.88%)	1,211 (78.59%)	42 (2.73%)	818 (53.08%)
HAI (n = 6,401)													
Sensitive	1,769 (27.64%)	2,078 (32.46%)	1,091 (17.04%)	1,260 (19.68%)	1,680 (26.25%)	1,310 (20.47%)	1,949 (30.45%)	2,182 (34.09%)	1,596 (24.93%)	2,512 (39.24%)	1,225 (19.13%)	4,607 (71.97%)	1,575 (24.60%)
Intermediate	1,101 (17.20%)	479 (7.48%)	650 (10.15%)	795 (12.42%)	762 (11.90%)	1,198 (11.72%)	825 (12.89%)	292 (4.56%)	1,699 (26.54%)	1,326 (20.72%)	160 (2.50%)	837 (13.08%)	1,268 (19.81%)
Resistant	3,531 (55.16%)	3,844 (60.05%)	4,660 (72.80%)	4,346 (67.90%)	3,959 (61.85%)	3,893 (60.82%)	3,627 (56.67%)	3,927 (61.35%)	3,106 (48.52%)	2,563 (40.04%)	5,016 (78.36%)	957 (14.95%)	3,558 (55.59%)

## Discussion

The AST findings of 7,942 adult patients with positive culture reports for *K. pneumoniae* were gauged. All types of clinical samples were considered for analysis in this retrospective study. Out of 50,057 samples positive for bacterial growth, 7,942 (15.87%) were positive for *K. pneumoniae*. Blood samples contribute to the majority of the positive culture reports. The study population comprised 4,948 (62.30%) males and 2,994 (37.70%) females. The participants’ average age was 53.50 (39.75-63.25) years. A total of 3,523 (44.36%) participants were admitted to the ICU. The incidence of HAI was around four times higher than that of CAI. The MDR cases were more prevalent in ICU patients (2,460, 30.97%) than in non-ICU patients (1,958, 24.65%). Tigecycline (5,940, 74.79%) demonstrated the maximum efficacy against *K. pneumoniae* isolates, followed by gentamicin (3,133, 39.45%), meropenem (2,727, 34.34%), and piperacillin-tazobactam (2,576, 32.44%). Ciprofloxacin (6,227, 78.40%), cefuroxime (5,809, 73.14%), and ceftriaxone (5,397, 67.96%) were the drugs against which *K. pneumoniae* isolates were highly resistant. The drug resistance was the least against tigecycline (999, 12.57%). Male preponderance and similar AST patterns of *K. pneumoniae* isolates had been observed in our earlier research paper [27]. Our AST findings matched the studies by Sharma et al. [23] and Kaur et al. [24].

Like other pathogens in the ESKAPE group,* K. pneumoniae* causes multiple infections like pneumonia, respiratory tract infection (RTI), UTI, skin and soft tissue infections, and meningitis [5]. The risks of morbidity and mortality due to these infections are higher for critically ill patients in the ICUs. Excessive and irrational usage of broad-spectrum antibiotics and India’s enormous population might have fuelled the MDR bacteria across the country [23,28,29]. In *K. pneumoniae*, the *blaKPC* genes exist mainly on plasmids, reducing susceptibility to nearly all beta-lactam antibiotics [30]. Age, infection source, underlying disease, polypharmacy, duration and frequency of antibiotics, non-compliance with hospital antimicrobial policy, and production of biofilms influence the incidence of CAI, HAI, and AMR [31,32].

The subgroup analyses yielded a few observations that differed from the study population's findings. The patients with HAI had similar AST patterns as the whole study population. For ICU patients, sensitivity was higher for meropenem than for gentamicin and piperacillin-tazobactam. For non-ICU patients, piperacillin-tazobactam was more effective than meropenem. The sensitivity of *K. pneumoniae* isolates towards cotrimoxazole was higher among those with CAI. However, the resistance against ciprofloxacin, cefuroxime, and ceftriaxone remained the highest for the entire study population and all four subgroups.

The pluses of our study were data analysis for ICU and non-ICU patients, those with CAI and HAI, and data interpretation via mosaic plot and chord diagrams. Our study could have been improved regarding certain aspects. First, the data was collected only from the microbiology laboratory. Hence, the AST findings could not be correlated with the prescribed antimicrobials. We could not trace the duration of stay or the outcome of the patients. Second, we could not perform whole-genome sequencing (WGS) because of the large study population and retrospective design. Third, we excluded *Klebsiella* species other than *K. pneumoniae* due to their low incidence. Fourth, we could not assess the impact of diagnosis, comorbidities, and concomitant medications on AST findings.

## Conclusions

The majority of samples positive for *K. pneumoniae* were obtained from blood, urine, and ETT. The incidence of HAI was four times that of CAI. MDR cases were more prevalent in ICUs. *K. pneumoniae* isolates showed high susceptibility towards tigecycline, gentamicin, meropenem, and piperacillin-tazobactam. The maximum resistance was seen against ciprofloxacin, cefuroxime, and ceftriaxone. We recommend prospective studies to determine the genetic association of growing antimicrobial resistance among *K. pneumoniae* strains.
